# Escape Room in Nursing Fundamentals Course: Students’ Opinions, Engagement, and Gameful Experience

**DOI:** 10.3390/nursrep15090343

**Published:** 2025-09-19

**Authors:** Dragana Simin, Aleksandra Plećaš Đurić, Branimirka Aranđelović, Dragana Živković, Dragana Milutinović

**Affiliations:** Department of Nursing, Faculty of Medicine, University of Novi Sad, 21000 Novi Sad, Serbia; aleksandra.plecas-djuric@mf.uns.ac.rs (A.P.Đ.); branimirka.arandjelovic@mf.uns.ac.rs (B.A.); dragana.zivkovic@mf.uns.ac.rs (D.Ž.)

**Keywords:** game-based learning, education, escape room, nursing students

## Abstract

**Background/Objectives:** During the past decade, incorporating innovative teaching strategies for active learning, such as the use of escape rooms (ERs), has effectively contributed to the acquisition of the necessary skills. This study aimed to assess students’ opinions, engagement, and gameful experience, and to analyse the impact of engagement and gameful experience on students’ opinions about ER activity. **Methods:** This descriptive-analytical, quantitative, and interventional cross-sectional study was conducted among first-year nursing students enrolled in the Nursing Fundamentals course. The ER activities took place in a faculty classroom. The measure included a questionnaire for assessing students’ opinions about ER activity, engagement while learning through play, and the Gameful Experience Scale. **Results:** The students reported very positive opinions on the outcomes of escape room activities. According to the students’ perception, solving puzzles required a high level of cognitive, emotional, physical, and other engagement. The experience of learning through play contributed to their increased enjoyment, absorption, and creative thinking, with a low level of negative effects and dominance. Enjoyment, immersion, and creative thinking during the gameful experience explained 49.0% of the variance in students’ opinions on ER activity. **Conclusions:** ER enabled students to consolidate knowledge from various fields within one lesson, encouraging them to be highly engaged and think creatively, giving them a sense of enjoyment in learning and motivation for further learning.

## 1. Introduction

Today’s increasingly dynamic and complex healthcare system imposes the need for continuous improvement of professional competencies in the preparation of the nurses of the future. Therefore, to develop professional, personal, and organisational skills, it is necessary to apply innovative teaching strategies in nursing education [1]. To achieve this, nurse educators must also focus on facilitating the development of students’ critical thinking skills. This is supported by the fact that nursing practice in the current healthcare setting is increasingly confronted by situations where nurses must make rational and quick decisions by setting priorities based on relevant evidence [2,3].

At the same time, nursing education is strongly influenced by technological progress and new pedagogical approaches, which shape how knowledge is delivered and acquired. In this context, innovative teaching and learning strategies are required to address the evolving needs of students and prepare them for future professional assignments [4].

Game-based learning (GBL) has emerged as one such strategy. It aligns with key theoretical perspectives, including Knowles’ principles of adult learning, which emphasise self-direction, relevance, and the practical application of knowledge, and Kolb’s experiential learning theory, which underscores the role of active experimentation and reflection in consolidating learning [5]. GBL also resonates with constructivist approaches, in which learners build knowledge through interaction, collaboration, and problem-solving in authentic contexts. Through these theoretical lenses, GBL is understood not only as a motivational tool but also as a pedagogical strategy that fosters deeper cognitive engagement and the integration of knowledge across disciplines [5,6].

Nowadays, nursing students appreciate and embrace the implementation of innovative teaching and active learning strategies such as GBL. GBL is a strategy that improves their satisfaction and the sense of joy experienced while investing mental effort in the learning process [7]. Over the last five years, GBL has become a trend in health professions education, suggested by an almost tenfold increase in scientific publications [5]. Therefore, an escape room (ER) represents an active T&L strategy, which integrates adult learning principles in addition to GBL [8,9]. ER activities are “bringing time-constrained authentic work settings or external world situations into the classroom”; in addition to enhancing students’ engagement, students must communicate and interact with each other to successfully complete their mission ([10], p. 11). However, using ER as an educational strategy for learning has a pretty short history [9].

In recent systematic reviews, Quek et al. [11] emphasise the importance of describing and evaluating the implementation of various educational resources, thereby providing educators with valuable insights into effective practices for implementing and assessing this educational strategy. Similarly, González-de la Torre et al. [12] emphasise that although ERs are increasingly prevalent in education, they have not yet been widely applied in nursing education across many countries. They further highlight the importance of expanding their use in different contexts and evaluating their effectiveness. Therefore, this study’s ER implementation and evaluation provide additional empirical evidence grounded in educational theory to inform educators and researchers.

## 2. Background

Traditional T&L strategies allow nursing students to identify solutions to problems in the future work environment based on what they have learned. However, all the GBL activities, especially ER activities, also provide real practice opportunities for nursing students to explore problems within a safe classroom setting within a specified time limit [3,8,10]. Thus, this will enable students to consolidate their prior knowledge and develop profession-specific competencies, attitudes, and motivation for learning [2]. Additionally, ER activities contribute to the development of collaboration, persistence, and adaptability, which are especially important for first-year university students transitioning from secondary education and adjusting to teaching and learning (T&L) in higher education [13].

The general premise is that implementing ER activity, as with any innovative educational strategy, should be accompanied by various evaluation methods [10]. The literature reflects that the evaluation of nursing ER activity, organised according to Kirkpatrick’s learning evaluation model, was often focused on the first level or students’ reactions to this educational strategy [11]. Although student opinions and perceptions of engagement represent the basic evaluation level and do not provide a comprehensive view of the effectiveness of any educational activity, they are very useful for modifying educational strategies [14]. Overall, nursing students have a positive opinion regarding using an ER as an increasingly popular teaching strategy [9,10,12,15]. It has also been shown that ERs used in nursing education positively affected nursing students’ educational outcomes [16]. Namely, according to students’ opinions on ER activities, it is not only that they make the learning process enjoyable, but they also have beneficial effects. Solving puzzles and accomplishing tasks to break out help them consolidate existing knowledge and skills, motivating them to learn even though they have much time before the exam [17]. Activities in ER encourage/initiate cognitive, emotional, and physical activities that promote student engagement in teaching [18]. At the same time, studies presenting various designs of ER activities to GBL as a basic teaching and learning strategy have shown that nursing students exhibit higher levels of enjoyment, absorption, creative thinking, and activation, with fewer negative effects [2,16,19].

Due to the COVID-19 pandemic disruptions during the academic year, students could not gain clinical experience. As an innovative and motivational strategy, the ER has been identified as a potentially appropriate teaching option. Namely, due to prescribed anti-epidemic measures for this academic year, most lectures were conducted online and only occasionally in the classroom [11,12,20,21]. Therefore, to achieve outcomes defined by the Nursing Fundamentals course for first-year nursing students, we implemented ERs through in situ practical training, utilising available resources such as space, time, and equipment. Although conducting the ER in a virtual environment could have offered advantages—particularly by enabling simultaneous participation of all students while maintaining physical distance—we decided to implement the ER on-site in the faculty skill lab. This decision was partly influenced by financial limitations, as our institution, located in a low-income country, lacked access to advanced digital tools and a learning platform capable of supporting virtual ER activities. Technical barriers also posed a concern, including the reliability of internet connections and the quality of devices available to students at home. Most importantly, however, the on-site ER allowed us to foster social interaction, promote direct teamwork with active participation of all students, and gain immediate insight into student activities.

The aims of this study were (a) to assess nursing students’ opinions, (b) to evaluate nursing students’ engagement, (c) to examine nursing students’ gameful experience during ER activities, and (d) to analyse how their engagement and gameful experience influenced opinions about ER activity.

## 3. Materials and Methods

### 3.1. Study Design

Descriptive-analytical, quantitative, and interventional cross-sectional study designs were conducted in May 2021.

### 3.2. Sample

A purposively selected sample comprised all first-year nursing students of the Faculty of Medicine of the University of Novi Sad (Serbia) in the 2020/21 academic year. Of the total number of students (N = 87), 16 were prevented from attending on-site classes (positive for SARS-CoV-2 or were in self-isolation due to contact), whereas 6 students refused to participate in the study.

### 3.3. Development and Implementation of the Escape Room

#### 3.3.1. Design and Rules

Puzzle contents were related to different fields of Nursing Fundamentals. The learning objectives of the ER were aligned with the Nursing Fundamentals course objectives, aiming to (i) consolidate knowledge of nursing procedures and patient care, (ii) strengthen teamwork and communication skills, and (iii) encourage critical thinking and problem-solving in time-limited scenarios. According to general recommendations, we did not use activities unknown to the students in the puzzles [1]. The design process ER activity followed the iterative ER design-based approach proposed by Eukel and Morel [22]. After creating the initial version of the ER, an expert panel of five nurse educators specialising in Nursing Fundamentals was established. After a consensus was reached among experts on the content, ER design, and the availability of resources for implementing escape rooms, a pilot study was conducted.

This pilot study was conducted two weeks before the main study implementation and included ten final-year nursing students divided into two groups. The inclusion criteria for the pilot study were voluntary consent, study year, and passing the exam in the subject where the ER is implemented.

Results from the pilot study indicated that the initially designed ER activities had some weaknesses. It was determined that 45 min was not enough for the limited time frame. Accordingly, the first group solved the puzzle within 59 min and the second within 56 min. Students in both groups then suggested that positioning a patient with pressure ulcers took a significant time (15 to 20 min). Also, they reported that incorporating available supplies and equipment within this puzzle did not enable them to solve it correctly. The students also pointed out that the written instructions for solving two puzzles were unclear in the “hints and guidelines”. In both cases, these were instructions on how to find hidden objects in the classroom. Since it was impossible to completely redesign the classroom to fit the needs of the ER, students found it challenging to locate the necessary objects in the closets, which were “stored with material” (containing resources for training in several nursing subjects).

After considering all the suggestions, the ER activity has been altered. Consequently, the patient positioning puzzle was not included in the final list of puzzles.

**Figure 1:**
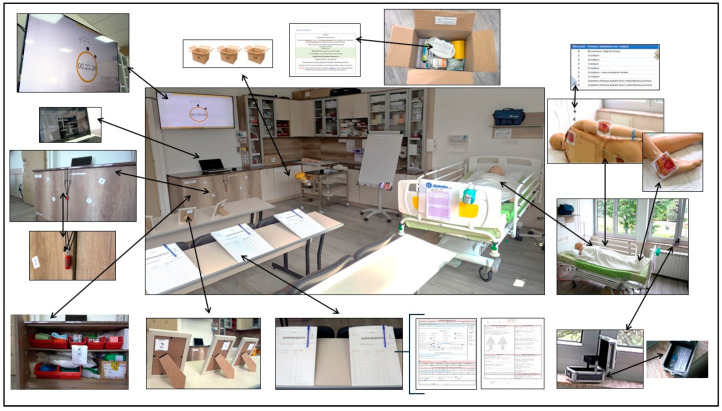
Faculty SkillLab and supplies for ER activity.

In order to complete the puzzles, the available classroom resources were mainly used, whereas the authors of the present study were obliged to provide supplies such as picture frames, boxes, keys, and padlocks (a total of approximately 60 EUR), Figure 1.

#### 3.3.2. Implementation

For the main study, students who met the inclusion criteria were divided into 16 groups, each containing 4 to 6 students. All of them received general instructions previously written on the classroom front door. Then, after entering the classroom, detailed information was provided on reasons, importance, and guidelines for conducting ERs. A nursing educator was also present during ER implementation, having an active role in all the groups. However, after the introductory information and the beginning of the activity, the participants could consult the educator only once. However, they did not stop the timer when providing additional information or instructions to solve the puzzle.

An animated stopwatch was set up on the LCD monitor to count down the time (45 min). The first puzzle required matching the definition of nursing to its author (photo in the frame). The number on the back of the picture frame indicated one of the three patient documents where the next puzzle was. To solve it, students had to assess the risk of pressure ulcer development based on the data obtained from the Nursing Care Plans by entering the data into the Braden scale. The calculated score was the code needed in the next puzzle, in which students within the team had to share their tasks. One part of the team calculated the flow rate of the prescribed volume of solution to be infused, whereas the other inserted a peripheral venous cannula on the training arm. The calculated flow rate directed them to the following location—the lockbox containing a text puzzle with instructions on inspecting a patient’s skin. When turning the “patient” (learning model), students could detect photos showing various stages of pressure ulcers. By fitting the puzzle pieces together according to the instructions, a four-digit code was obtained, and the numbers together unlocked the padlock needed to open the closet where the puzzle was located.

**Figure 2:**
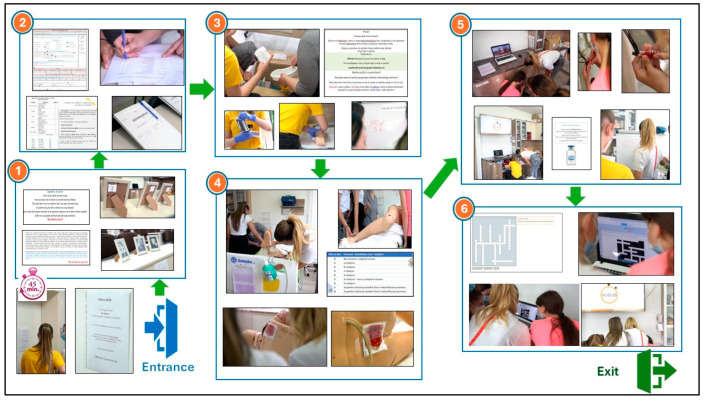
Students’ activity from entrance to exit from “Room”.

Furthermore, to solve the new puzzle, students were expected to examine the patient’s documentation, where the doctor prescribed a therapeutic drug dosage that was less than the available pharmaceutical dosage. Finally, the calculation-based number was the code that unlocked the laptop with the final puzzle, which included 11 crossword puzzle questions. After successfully solving the crossword puzzle, “the door to escape opened” (Figure 2).

Immediately after the students “escaped”, the teacher debriefed with them according to the model recommended by Davis et al. [1].

To increase students’ motivation to participate in the ER and prevent passing on the codes to other groups, students from the fastest group were awarded 1 out of 10 maximum possible points earned for passing the subject. Then, students from the second-place group earned 0.7 points, whereas 0.5 points were given to those ranked in third place. After each group had at least 30 min of recess, they were taken out of the classroom so the surfaces could be disinfected, the classroom ventilated, and the workstations returned to their initial state. The exercise was carried out by three to four groups each day.

### 3.4. Instruments and Data Collection Methods

Socio-demographic questions determining the general characteristics of students included questions about gender, age, previous participation in ER classes, and playing recreational ER.

We used a questionnaire by Gómez-Urquiza et al. [17] to assess students’ opinions on escape rooms. The questionnaire comprised six items on a five-point Likert scale ranging from 1 = I strongly disagree to 5 = I strongly agree. A higher score indicates a more positive opinion about class activities.

A questionnaire created by Yosanovich [18] to evaluate students’ engagement in ER activities by adapting one of the most commonly used questionnaires to measure work engagement of the human spirit at work (created by May et al. [23]) was used. A 17-item questionnaire included four subscales (cognitive, emotional, physical, and other engagement). Students’ perceptions of engagement ranged on a five-point Likert scale from 1 = “low” to 5 = “high” engagement. Given that a score above average indicates a higher level of student engagement, reverse scoring in data processing was used on five statements due to negative connotations (2, 5, 9, 10, and 14).

The Gameful Experience Scale (GAMEX) by Eppmann et al. [24] assessed the game-based learning experience. The scale contained 27 items, divided into six dimensions: enjoyment, absorption, creative thinking, activation, absence of negative effect, and dominance. The items are evaluated using a five-point Likert scale ranging from 1 = never to 5 = always. The dimensions cover various aspects of the experience: Enjoyment (6 items) relates to fun and satisfaction, Absorption (6 items) to immersion and focus, and Creative Thinking (4 items) to stimulate imagination or creativity. Activation (4 items) reflects the sense of activity during the game, while Absence of Negative Effects (3 items) addresses the avoidance of unpleasant emotions such as frustration. Finally, Dominance (4 items) captures the degree of confidence, autonomy, and control students experienced in the game. Additionally, in the text-based instructions for obtaining the best response to questions, students were informed that the game can be defined as a play-based learning activity during the ER.

The questionnaires assessing students’ opinions and engagement in the ER, as well as the GAMEX scale, were translated into Serbian using the standard forward–backwards translation procedure [25]. A panel of experts, consisting of four nurse educators and three master’s students in nursing, confirmed their face validity, with affirmative responses of 96% for the questionnaires on students’ opinions and engagement in ER, and 97% for the GAMEX scale, thus supporting their acceptability [26]. In addition, the reliability of all instruments was assessed using Cronbach’s alpha coefficient, which was 0.76 for the questionnaire on students’ opinions, 0.71 for the questionnaire on engagement in the ER, and 0.87 for the GAMEX scale.

Paper-based versions of all the questionnaires were distributed in the classroom immediately after the ER.

### 3.5. Ethical Considerations

The present study obtained approval from the Ethics Committees of the Faculty of Medicine, University of Novi Sad, under reference number 01-39/35/1 (date of approval 15 April 2021). After receiving research-related information, by noting that consent, completion, and results of the questionnaire would not affect their success in any subject, students who decided to participate in the study provided written informed consent following the Declaration of Helsinki.

### 3.6. Statistical Data Processing

Statistical analysis was performed using SPSS software version 26.0. Characteristics of numerical attributes were presented as frequencies and percentage values, i.e., mean values (M) and standard deviation (SD). Normality of the data distribution was assessed using the Kolmogorov–Smirnov test. As the data met the normality assumption, independent samples *t*-tests were conducted to determine whether significant differences existed in students’ socio-demographic characteristics. Effect sizes (Cohen’s d) were also calculated to quantify the difference. The standard multiple regression analysis was used to analyse the impact of engagement and gameful experience on the nursing students’ opinion of ER activity. A *p*-value less than 0.05 was considered statistically significant in all analyses. The reliability of all the questionnaires was analysed using Cronbach’s alpha coefficient.

## 4. Results

### 4.1. Socio-Demographic Characteristics

The majority of the total number of students (N = 65) were females (87.7%). The average age of students was 19.9 years (SD = 2.5), with the youngest being 19 and the oldest 27 years. None of the students had attended classes using the ER methodology in their previous schooling. Seven students (10.8%) had previously played recreational ERs.

### 4.2. Playtime

**Figure 3:**
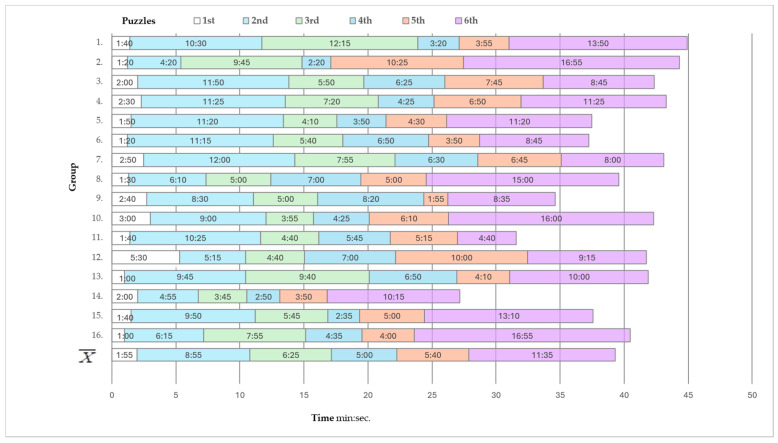
The average time each group spent solving each of these puzzles.

On average, it took 38.5 min to enter the classroom until exiting the escape room, i.e., by solving all the puzzles. The fastest group needed 27.1 min to escape, and the slowest group required almost 45 min. On average, it took students two minutes to solve the first puzzle and almost another ten minutes for the last crossword puzzle-solving (Figure 3).

### 4.3. Students’ Opinions About ER Learning Activities

Generally, students reported very positive opinions about ER activities (Table 1). Among students who did not play recreational ERs compared to students who played this game (*p* = 0.006), there were significantly more positive opinions about ER activities, which greatly increased their motivation and willingness to learn, although they had plenty of time to prepare for the final exam. Cohen’s d indicates a small effect size of the difference between these two groups of students. The results have also shown that according to students’ opinions for other items related to this characteristic and their gender, there were no statistically significant differences.

### 4.4. Student Engagement Questionnaire

According to students’ perceptions, the results in Table 2 indicate that ER activities required a high level of engagement in all subscales. The mean score by subscales was cognitive 4.41 ± 0.30, emotional 4.10 ± 0.49, physical 4.22 ± 0.43, and 4.55 ± 0.47 for other engagements.

Most items measuring cognitive engagement revealed that male students had higher engagement levels than female students. However, only the first item of this subscale showed a significant difference, with a large effect size (Cohen’s d = 1.06).

Regarding physical engagement, the mean score was 4.26 ± 0.43 in students who had not played recreational ERs before participating in this ER, whereas it was 3.83 ± 0.44 in those with prior ER experience. The difference between the mean score of students’ perception of their physical engagement level was statistically significant, with a large effect size (Cohen’s d = 0.99). Thus, unlike students without ER experience, all students with prior ER experience strongly agreed with participating in the exercise until it was finished (*p* = 0.000), and their feeling of working too hard during the activity was significantly milder.

The highest average score was recorded for the subscale of other engagements, which consisted of three questions focused on the students’ opinions about the importance of their work in contributing to activities in ER, where they were not afraid to be themselves and to express their opinions during engagement in the ER. Although male students, as well as those who had not previously played ER recreationally, had higher average scores on this subscale, compared to female students, i.e., those who played ER recreationally, these differences were not significant.

### 4.5. Gameful Experience Scale

The mean scores greater than three included the following four students’ gameful experience dimensions: enjoyment 4.81 ± 0.89, creative thinking 4.55 ± 0.88, absorption 3.59 ± 0.98, and activation 3.21 ± 0.81. The mean score on the dominance dimensions was 2.73 ± 0.89, indicating moderate student dominance during the ER activities. The lowest mean score of 1.23 ± 0.77 was found for the absence of a negative effect (Table 3).

The level of enjoyment in learning through play was significantly higher among male students, with a large effect size (Cohen’s d = 0.97). In addition, students who had previously played recreational ER enjoyed this educational ER significantly more than their counterparts (*p* = 0.001, Cohen’s d = 0.92).

### 4.6. Results for Standard Multiple Regression Analysis

The final model includes cognitive, emotional and other engagements, enjoyment in play-based learning experiences, immersion, and creative thinking, and explains 49.0% of the variance in students’ opinions on ER activity. Among all the variables, creative thinking makes the strongest unique contribution (Beta = 0.408), followed by enjoyment and students’ absorption in the ER activities, with the lowest contribution level (Table 4). Creative thinking as a dimension of gameful experience contributes statistically significantly and uniquely explains 8.2% of the variance of the students’ opinions about ER activity.

## 5. Discussion

In the present study, first-year nursing students’ engagement during the ER enabled them to quickly demonstrate and revise their knowledge in the different fields of Nursing Fundamentals. The evaluation after the ER aimed to analyse students’ opinions, engagement, and gameful experience, as well as the effects of students’ engagement in gameful learning experiences on their opinions of the ER activity.

In designing the ER activities, panel experts and students participating in the pilot study agreed that the puzzle content was adequate, but the time limit was inadequate. Playtime is an essential element of GBL integrated into every nursing ER because, per future professional tasks, nursing students often require adequate time-limited solutions [17]. However, a limited time to solve puzzles can lead to students’ frustration, dropping out of the game, and pointless attempts and mistakes, as failures are marked by not accomplishing the goals of the ER [27]. Therefore, after the pilot study, we redesigned the ER so that all groups successfully “escaped” in the allotted time in the final version. By ER design aims, all the groups solved the first puzzle in the shortest time. Namely, the first relatively simple puzzle aimed to help students get started with the other ER activities.

Planning, designing, and implementing ER activities in nursing education requires significant commitment from enthusiastic teachers [7,28]. Certainly, to make ER activities effective and achieve all the planned goals in the course context, as well as in applying any other GBL educational strategy, teachers must possess gamification literacy [29]. The advantage of ER in an on-site environment is that it does not require teachers to have advanced digital literacy [28]. In our study, the teacher present while solving the puzzle could, according to the defined rules, be consulted once by the students. The questions and doubts that were the reason for the consultation mostly related to puzzles that contained nursing calculations, dilemmas about the formula and application of mathematical operations, finding key data in the patient documentation necessary for assessing the risk of pressure ulcers (according to the Braden scale), and in crosswords, choosing the correct term. However, the overall teacher role was deliberately minimal to encourage autonomous puzzle-solving and teamwork. At the same time, in the short debriefing after the ER, in addition to expressing satisfaction and the general assessment that they enjoyed themselves during the ER, these doubts were the main responses to the question of what they would have done differently.

Our students’ opinions about ER activities are very positive. The results of students’ opinion analysis in this and several previous studies clearly show that enjoyment during learning is one of the benefits of an ER [27,28,30,31,32,33,34,35,36]. Given that enjoyment, while learning, encourages the whole learning process, it was this benefit that, even before the pandemic, suggested an ER as a promising innovative strategy trend [20]. Additionally, in the educational context, ER activities enable nursing students to demonstrate their knowledge [8] and to recognise T&L gaps for both themselves and their teachers [37]. Furthermore, our students believe that ER activities enabled them to recall and apply previous knowledge, helping them master the educational material and pass the exam in the Nursing Fundamentals subject. Moreover, ER motivated them to engage in further learning, although they had much time until the exam. These results are consistent with the results of studies where an ER has been applied to nursing students in Adult Nursing 1 [17], Fundamentals Nursing II—parenteral drug administration [33], History, Theory and Methods of Nursing II [34], Community Care [2], Mental Health [28], Sexual and Reproductive Health Care [35], and Anatomy [27].

The data from this study showed that solving ER puzzles required students to have a high level of cross-domain engagement. The highest average score was for the other types of engagement subscale, where almost all students stated that they were not afraid to express their opinions and would not hesitate to have their own. As this may indicate, an ER is experienced as a psychologically safe environment, representing these claims as their experience of psychological security [18,23,33]. The regulation of emotions and processing of emotional information related to puzzle-solving activity are also associated with the social interactions of group members, aiming to solve all the puzzles and escape the room [38]. Our students’ perceptions of ER activities have shown that they significantly enhance their emotional engagement. Namely, in addition to excitement, most felt a total commitment to the ER and the team’s emotional attachment. However, their feelings were not influenced by how well the ER activities were completed. All ER activities are designed to enhance students’ cognitive engagement. Therefore, if cognitive engagement is not achieved, the ER will probably not be effective in helping students achieve their learning goals [39]. Therefore, if students do not have optimally challenging tasks or experience negative emotions, such as frustration, they will be less engaged and committed to the task, and students’ learning in achieving goals will be negatively affected [40]. In the pilot study, we focused on potential triggers for students’ frustration. So, a higher student engagement on a cognitive level can be explained by optimally more challenging tasks in the final ER activities.

ER activities integrate components of gamification that appeal to today’s students, also known as Millennials and Generation Z [41,42,43]. In line with this view are the high average grades of our students for the first three dimensions of the GAMEX scale, which indicate that during learning through play, they had a strong experience of enjoyment, absorption, and creative thinking in the activities of this ER. According to Wynn [20], fostering student satisfaction during the pandemic era was considered crucial for promoting resilience to stress in disrupted T&L environments. The absorption-related dimension of the GAMEX scale includes items related to immersion, flow, or presence during gameplay activities [24]. Student immersion during ER activities is vital for achieving learning outcomes through this strategy [10,33]. Consistency in ER design, where all of the puzzles and equipment within the ER are relevant to the content-based theme and purposeful, can prevent cognitive dissonance, fostering complete immersion and, therefore, students’ engagement [44]. In well-designed ERs, in addition to a consistent narrative, content-based puzzles, locks, and clues are tools for creating learning environments through play-based learning experiences that contribute to better content mastery [28,34,45]. High levels of our students’ immersion in the ER activities were probably achieved due to the game site, the well-known environment, and the fact that we eliminated all the vagueness and ambiguities detected in the pilot study. To escape from the room within a set time limit required our students and those in previous studies to think effectively through creative puzzle-solving [2,32].

At the same time, low levels of anxiety, hostility, and frustration regarding using ER activities in the dimension of negative effects were observed among our students and nursing students in previous studies [16,35,46]. The authors of the GAMEX scale indicate that a high level of gameful experience is associated with two opposing emotional dimensions: a high level of enjoyment and the absence of negative or a few negative effects [24]. Activities that maximally encourage opportunities for collaborative activities are essential to minimise the negative effects of mutual competition, such as demotivating students who perceive themselves as incompetent during the game [40]. Activities of various designs lead to ER experiences that manifest in different ways, involving the experience of enjoyment almost always. In contrast, the sense of dominance varies with design, so competitive activities, e.g., strengthening leadership, strongly contribute to strengthening dominance, and participants feel more dominant [24].

In contrast, ER activities that promote social interaction and collaborative problem-solving contribute to students’ descriptions of their experience as collaborative and helpful in appreciating and improving their teamwork skills [21,47]. Accordingly, in line with the nature of our ER, it is not surprising that our students also reported a lower level of dominance, i.e., they did not feel dominant, influential, or autonomous. However, at the same time, they felt confident during the ER activities.

Analysing gender-related data derived from the GAMEX scale, our male students had significantly higher mean scores in the dimension of dominance than female students. Similar results have been reported in nursing students in several recent studies [2,16,28,36]. Our results also indicated that ER activities were more interesting and appealing to male students regarding cognitive engagement, whereas female students reported stronger feelings of belonging to the team. In addition, prior literature has shown that male students are more competitive [36] and tend to score higher on personal values such as Power, Achievement, and Hedonism [48].

This educational ER was our students’ first learning experience with an educational ER, among whom only seven had prior experience in recreational ERs. This experience may have helped them enjoy the game more and put in less effort than students without ER experience. Veldkamp et al. [49] pointed out that regarding students’ beliefs, even if the content of the ER changes, their way of thinking remains the same, so that the previous ER experiences might help them with each new ER.

Educational ER is suitable for today’s students because they prefer active engagement, and solving puzzles requires students to think critically and creatively [33,36,50]. The results of the regression analysis of this study confirmed these claims. Namely, a play-based learning experience encouraged students’ creative thinking when solving puzzles, resulting in a more significant contribution to students’ positive opinions on ER activity. Mullen and Seiler [51] indicate that ER activities engage students in obtaining creative problem-solving solutions. At the same time, they conclude that critical thinking skills, which are developed during the ER, will help students use evidence and know how to justify their decisions in future professional tasks.

### Limitations

This study has some limitations. First, the question arises as to whether we have adequately motivated students not to disclose any information, codes, or clues to the following groups. In addition, several studies provided data on the number of students who had previous experience with ER; however, the results overview and subsequent discussion were not commented upon, which led us to compare different groups unrelated to nursing. Finally, a limitation of this study is that it did not assess the subsequent impact of the ER on students’ academic performance or clinical competencies. Although students’ engagement and perceptions were assessed, monitoring exam results or other objective learning outcomes would have provided a more comprehensive understanding of the effectiveness of this teaching–learning strategy.

## 6. Conclusions

This study adds to the growing body of literature on game-based learning in nursing education by providing empirical evidence on the effectiveness of ERs in simultaneously enhancing multiple dimensions of student engagement, learning, and professional development. While previous studies have mainly described ERs as innovative and enjoyable, our findings extend this knowledge by demonstrating that students also perceive them as psychologically safe environments that foster both cognitive and emotional engagement. In addition, this study highlights how learning through play in an escape room setting promotes enjoyment, absorption, and creative thinking, with minimal negative effects. These findings support the integration of escape rooms as a pedagogical strategy that motivates students and consolidates knowledge across disciplines within a limited timeframe. Notably, the results underscore the value of escape rooms as an alternative learning approach when traditional clinical practice opportunities are restricted, as was the case during the COVID-19 pandemic.

## Figures and Tables

**Table 1:** An average score of each item in the student opinion questionnaire: differences related to students’ gender and prior experience playing recreational ER.

Item	All Students	Gender				Prior Experience Playing Recreational ER			
		Male	Female	t	*p*	Yes	No	t	*p*
	M ± SD	M ± SD	M ± SD			M ± SD	M ± SD		
Playing the game helped me learn the subject	4.49 ± 0.53	4.80 ± 0.44	4.47 ± 0.53	1.575	0.176	4.50 ± 0.54	4.49 ± 0.53	0.037	0.971
I enjoyed playing the game	4.95 ± 0.27	5.00 ± 0.00	4.95 ± 0.28	0.387	0.700	5.00 ± 0.00	4.95 ± 0.28	0.428	0.670
I think the game will help me in the exam	4.32 ± 0.73	4.60 ± 0.54	4.30 ± 0.74	0.880	0.382	4.17 ± 0.75	4.34 ± 0.73	0.547	0.586
I remembered and applied subject knowledge during the game	4.68 ± 0.53	4.60 ± 0.54	4.68 ± 0.53	0.333	0.740	4.67 ± 0.51	4.68 ± 0.53	0.049	0.961
There should be more games of this type in nursing studies	4.86 ± 0.39	5.00 ± 0.00	4.85 ± 0.40	0.823	0.413	5.00 ± 0.00	4.85 ± 0.40	0.911	0.366
The game has motivated me to study further, although the exam is still 6 weeks away	4.37 ± 076	4.60 ± 0.54	4.35 ± 0.77	0.702	0.485	4.17 ± 0.75	4.39 ± 0.76	2.876	0.006
Total	4.64 ± 0.34	4.77 ± 0.19	4.62 ± 0.36	0.871	0.387	4.59 ± 0.21	4.64 ± 0.36	0.322	0.749

**Table 2:** Average score of each subscale in the Student Engagement Questionnaire: differences in relation to students’ gender and their prior experience playing recreational ER.

Subscale	All Students	Gender				Prior Experience Playing Recreational ER			
		Male	Female	t	*p*	Yes	No	t	*p*
	M ± SD	M ± SD	M ± SD			M ± SD	M ± SD		
Cognitive	4.41 ± 0.30	4.50 ± 0.42	4.40 ± 0.42	0.496	0.62	4.44 ± 0.29	4.40 ± 0.42	0.208	0.836
Emotional	4.10 ± 0.49	4.10 ± 0.63	4.09 ± 0.48	0.018	0.986	4.00 ± 0.42	4.10 ± 0.5	0.504	0.60
Physical	4.22 ± 0.43	4.35 ± 0.38	4.21 ± 0.45	0.665	0.508	3.83 ± 0.44	4.26 ± 0.43	2.344	0.022
Other	4.55 ± 0.47	4.67 ± 0.46	4.54 ± 0.47	0.562	0.576	4.44 ± 0.54	4.56 ± 0.46	0.602	0.549

**Table 3:** Average score of each dimension in the Gamex Experience Scale: differences by students’ gender and their prior experience playing recreational ER.

Dimensions	All Students	Gender				Prior Experience Playing Recreational ER			
		Male	Female	t	*p*	Yes	No	t	*p*
	M ± SD	M ± SD	M ± SD			M ± SD	M ± SD		
Enjoyment	4.81 ± 0.89	4.93 ± 0.09	4.78 ± 0.40	2.327	0.029	4.86 ± 0.27	4.78 ± 0.40	0.456	0.650
Absorption	3.59 ± 0.98	3.90 ± 0.27	3.67 ± 1.04	0.493	2.624	3.89 ± 1.18	3.66 ± 1.00	0.516	0.607
Creative thinking	4.55 ± 0.88	4.60 ± 0.45	4.52 ± 0.59	0.305	0.761	4.54 ± 0.46	4.52 ± 0.60	0.081	0.935
Activation	3.21 ± 0.81	3.35 ± 0.57	3.31 ± 0.58	0.801	0.426	3.50 ± 0.94	3.11 ± 0.53	1.571	0.121
Absence of negative affect	1.23 ± 0.77	1.27 ± 0.36	1.25 ± 0.60	0.061	0.952	1.72 ± 1.47	1.20 ± 0.40	0.863	0.427
Dominance	2.73 ± 0.89	2.25 ± 0.66	2.73 ± 0.86	1.216	0.229	3.75 ± 0.95	3.53 ± 0.42	0.803	0.425

**Table 4:** Standard multiple regression analysis.

	Unstandardised Coefficient		Standardised Coefficient	t	*p*	Correlations Part
	ß	SE	Beta			
Constant	8.013	2.789		2.873	0.006	
Cognitive engagement	0.122	0.102	0.140	1.198	0.236	0.107
Emotional engagement	0.040	0.121	0.036	0.332	0.741	0.030
Engagement—other	0.156	0.175	0.099	0.891	0.377	0.080
GAMEX—Enjoyment	0.226	0.118	0.238	1.908	0.061	0.170
GAMEX—Absorption	0.009	0.040	0.026	0.232	0.818	0.021
GAMEX—Creative Thinking	0.384	0.119	0.408	3.214	0.002	0.287

Model ANOVA: F = 11.241, *p* = 0.000.

## Data Availability

The data are available from the authors upon request.

## Public Involvement Statement

No public involvement in any aspect of this research.

## Guidelines and Standards Statement

This manuscript was drafted against the STROBE for a cross-sectional study.

## Abbreviations

The following abbreviations are used in this manuscript:

ER	Escape Room
T&L	Teaching and learning
GBL	Game-based learning
GAMEX	Gameful Experience Scale
M	Mean values
SD	Standard deviation
